# Methods used to account for caregivers’ sex and gender within studies examining the financial burden of caregivers of children and adolescents : Results from a scoping review

**DOI:** 10.2147/CEOR.S443077

**Published:** 2024-01-26

**Authors:** Jason Robert Guertin, Mahée Gilbert-Ouimet, Michèle Dugas, Valérie Carnovale, Laura Jalbert, Olha Svyntozelska, Juliette Demers, Léonie Matteau, Frédéric Bergeron, Annie LeBlanc

**Affiliations:** 1 Centre de recherche du Centre de recherche du CHU de Québec-Université Laval, Quebec City, Quebec, Canada; 2 Faculty of Medicine, Université Laval, Quebec City, Quebec, Canada; 3 Centre de recherche en organogénèse expérimentale de l’Université Laval/LOEX, Quebec City, Quebec, Canada; 4 Department of Health Sciences, Université du Québec À Rimouski, Levis, Quebec, Canada; 5 VITAM Research Center on Sustainable Health, Quebec Integrated University Health and Social Services Center, Quebec City, Quebec, Canada; 6 Bibliothèque-Direction des services-conseils, Université Laval, Quebec City, Quebec, Canada

**Keywords:** scoping review, caregivers, financial burden, sex and gender considerations, methods

## Abstract

**Background:**

Interest in the financial burden of informal caregivers has been growing. Unfortunately, it remains unclear which method(s) should be used when quantifying this burden.

**Purpose:**

We conducted a scoping review aimed at identifying which methods have been used to conduct such work and quantified their performance. We were also interested in examining how sex and gender considerations were considered within selected studies.

**Data Sources:**

Using a standardized approach, we identified studies published between 2012 and 2022 that aimed to document the financial burden of caregivers to child and adolescent patients. Our search strategy was applied to the MEDLINE, Embase, CINHAL, and Academic Search Premier databases.

**Study Selection:**

Manuscript selection was performed by pairs of reviewers.

**Data Extraction:**

Data extraction was performed by one reviewer with a second reviewer performing quality control. Results were reported using a narrative approach.

**Data Synthesis:**

We identified 9801 unique citations, of which 200 were included in our review. Selected studies covered various disease area (eg, infection/parasitic diseases [n = 31, 16%]) and included quantitative (n = 180, 90%), qualitative (n = 4, 2%) and mixed study designs (n = 16, 8%). Most studies (n = 182, 91%) used questionnaires/surveys, either alone or in combination with other methods, to assess caregivers’ financial burden. Less than half (n = 93, 47%) of studies reported on caregivers’ sex and none reported on their gender.

**Conclusion:**

We conducted an unrestricted review of published studies examining caregiver’s financial burden which allowed us to identify general methodological trends observed in this literature. We believe this work may help improve future studies focusing on this important issue.

## Introduction

Chronic diseases affect 22–25% of children aged 0–18 years in North America.^1^,^2^ These diseases generally require a combination of care involving healthcare settings and families.^3^,^4^ Although caring for children is a routine role for parents, when a child suffers from a chronic disease, this role tends to be complexified and can generate a burden.^5^ This burden, experienced by unpaid individuals caring for a patient and/or a relative (hereby referred to as “informal caregivers”), has received growing interest over recent years.^6–10^ The informal caregivers’ burden refers to the level of multifaceted strain, responsibilities and limitations perceived by the informal caregivers, originating from conflicts between care needs and other duties of the caregiver.^11^,^12^ It has been associated with deteriorations of the emotional and physical health, social life, and financial status.^13^

There is interest from health economists and experts in health technology assessment to quantify the value of the informal caregiver’s economic burden.^14–18^ Depending on the patients and their condition, informal caregivers have been known to incur both direct (eg, spending for medical equipment and drugs) and indirect costs (eg, conducting unpaid household chores, taking unpaid leave of absence from work to attend a patient’s clinical appointment, seeking professional help such as psychologists to help coping with emotional strain, or even retiring early from work) due to their involvement with patients.^5–8^,^11^,^19–24^ For example, in the United States of America, an additional US$1377.60–US$9059.49 annually were spent by informal caregivers on medical expenses for children aged 0–18 years with chronic diseases such as asthma, diabetes, or epilepsy compared to children without these conditions.^25^

Much of the work that has been conducted in the area of informal caregivers’ economic burden has focused on quantifying the amount directly and indirectly spent by caregivers of patients suffering from specific diseases, including Alzheimer’s disease,^26–28^ oncology,^29–31^ and rare diseases.^32–34^ These studies indicated that the caregiver’s relationship with the patient influences the level of economic burden imposed.^35^ This is especially the case for individuals caring for underaged patients (eg, parents and guardians of children and adolescent patients), as these patients may be unable to provide financially for themselves, therefore shifting expenses to their caregivers. Furthermore, in situations where the child suffers from a chronic condition, it is not uncommon for the caregiver’s employment status to change, with some caregivers completely abandoning their employment to care for their child.^24^ Also, caregivers of children with health problems are themselves at increased risk of developing health problems, leading to additional expenses.^19^,^21^,^23^,^36–40^ These caregivers were for instance shown to have higher risks of chronic health problems, activity limitations, and depressive symptoms compared with caregivers of healthy children.^20^

The financial burden of caregiving for a child is further complexified by the fact that sex and gender are major determinants of the caregiving experience.^20^,^35^,^41^,^42^ The concept of sex, referring to a set of biological attributes assigned at birth, is often interchangeably used with the concept of gender, which refers to sociocultural characteristics evolving in time.^42^ Indeed, not only are females more likely than males (sex) to be informal caregivers, gendered characteristics traditionally ascribed with femininity have also been shown to increase the burden.^43–45^ For instance, while typically feminine personality traits such as being nurturing can influence the nature of care provided to the child (eg, being fully present, validating feelings, providing physical affection), feminine roles such as being the primary caregiver of a household can increase the amount of care provided^24^,^41^,^42^,^45^ and consequently lower working hours.^46^,^47^ In addition, females caregivers report higher levels stress, anxiety, fatigue, low self-esteem, and optimism compared with males caregivers, further increasing their burden.^46–49^ As such, any assessment of the economic burden of caregivers should account for sex (biological) and gender (sociocultural) considerations.

Methods to estimate this burden, especially regarding gendered roles, are lacking. Thus, our study aimed to identify and describe approaches and measures used to document the financial burden of informal caregivers. Specifically, the objectives of this study are to 1) identify approaches (eg, interviews, surveys) and measures (eg, validated questionnaires) that document one or more aspects of the financial burden of informal caregivers; 2) describe the performance (eg, response rate, aspects covered) of these approaches and measures; and 3) document to which extent considerations of sex and gender are addressed within these approaches and measures.

## Materials and Methods

### Study Design

We conducted a scoping review in accordance with the Joanna Briggs Institute Manual for Evidence Synthesis^50^ and followed the Preferred Reporting Items for Systematic Reviews and Meta-Analysis extension for Scoping Reviews (PRISMA-ScR) for its reporting (the PRIMA-ScR checklist is provided within Appendix 1).^51^

### Search Strategy and Eligibility Criteria

We report our eligibility criteria using the PCC criteria (participants, concept, context) framework. Participants: We included parents or legal guardians who provided care and support for dependents without direct remuneration (hereafter, caregivers). Concept: Economic burden of caregivers such as direct (eg, value of expenditure) and indirect costs (eg, value of labour, productivity losses) required to deal with a specific situation. In addition, the methods by which information on the economic burden was documented, which included both qualitative (eg, interviews) and quantitative (eg, questionnaires) approaches. Context: We chose a worldwide context, ie, no limits regarding countries or language. Our searches focused on published literature to identify and describe approaches and measures used in primary studies to document the economic burden of caregivers. Thus, commentaries, protocols, abstracts, theses, books, and grey literature were excluded. Our review protocol was registered on the Zenodo platform on March 31st, 2022.

An experienced information specialist developed the MEDLINE (Ovid), Embase, CINHAL (EBSCO), and Academic Search Premier (EBSCO) search strategies in consultation with the review team. We limited results to papers published between 2012 and 2022 in peer reviewed journals. Another information specialist peer reviewed the strategy according to the PRESS checklist.^52^ All searches were conducted on March 24th, 2022 (Appendix 2). Deduping was performed in the EndNote software (Clarivate Analytics, LLC) using the Bramer method.^53^

### Data Selection and Extraction

We developed standardized forms for study selection and data extraction and conducted pilot exercises with all reviewers. Study selection was performed by pairs of independent reviewers while extraction was performed by one reviewer, with a second reviewer performing quality control for all data extraction forms. We resolved discrepancies by discussion or by consulting with a senior reviewer. Extracted data included: 1) study characteristics (eg, country, study design, approaches used to measure economic burden of caregivers, sample size, response rate); 2) direct and indirect costs (eg, direct medical and non-medical costs, indirect costs such as absenteeism); and 3) sex and self-reported gender identity (eg, presence of variables and extent of use). We used the DistillerSR software to manage this review (DistillerSR. V.2.35. Evidence Partners; 2021).

### Data Synthesis

We report data using a narrative approach which includes tables and graphs demonstrating the study characteristics, direct costs (medical and non-medical) and indirect costs. Our data synthesis is focused on providing a descriptive summary of the approaches used to measure the economic burden of caregivers in addition to sex/gender dimensions in the included studies on this subject.

## Results

### Literature Search

**Figure 1:**
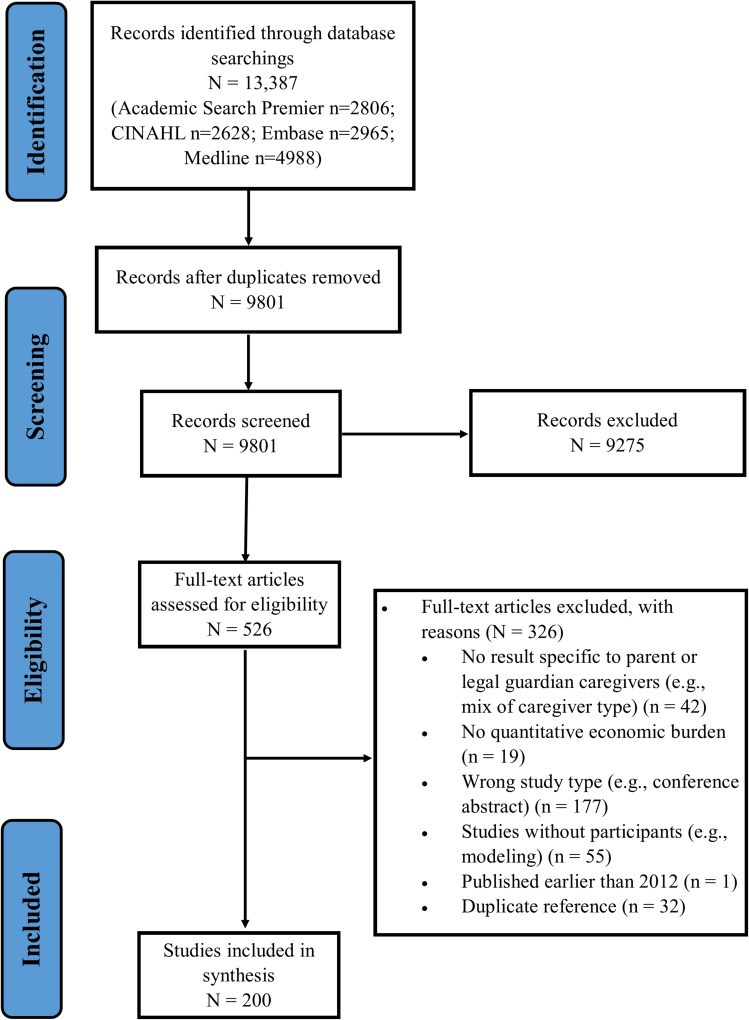
PRISMA flow-chart.

**Notes**: PRISMA figure adapted fromTricco AC, Lillie E, Zarin W, et al. PRISMA Extension for Scoping Reviews (PRISMA-ScR): checklist and Explanation. Ann Intern Med. 2018;169(7):467–473. Creative Commons.^51^

Our search strategies yielded a total of 9801 titles and abstracts following deduplication (Figure 1). We excluded 9275 references after the screening of titles and abstract. A total of 526 references were assessed for eligibility based on their full text. Ultimately, 200 articles were included in our review.^33^,^54–251^,^255^

### Characteristics of Included Studies

Table 1 provides a description of the studies included in our scoping review. The included studies were from a wide variety of countries, which we grouped into continents to simplify data synthesis. Studies were from North America (n = 55, 27.5%), Asia (n = 49, 24.5%), Europe (n = 41, 20.5%), Africa (n = 34, 17.0%), South America (n = 8, 4.0%), Oceania (n = 9, 4.5%) or multiple continents (n = 4, 2.0%). Most studies had a quantitative design (n = 180, 90.0%), followed by mixed methods (n = 16, 8.0%), and qualitative designs (n = 4, 2.0%).

**Table 1:** Characteristics of Studies Included in the Scoping Review

Study Characteristics	N (%) Total=200
**Continent**	
North America	55 (27.5)
Asia	49 (24.5)
Europe	41 (20.5)
Africa	34 (17.0)
Oceania	9 (4.5)
South America	8 (4.0)
Multiple^a^	4 (2.0)
**Study Type**	
Quantitative Study	180 (90.0)
Mixed Methods	16 (8.0)
Qualitative Methods	4 (2.0)
**Conditions of the Child or Adolescent Being Cared for by the Caregiver**	
Infections/Parasitic Diseases	31 (15.5)
Congenital Diseases	23 (11.5)
Gastrointestinal Issues	21 (10.5)
Respiratory Issues	21 (10.5)
Nervous System Diseases	20 (10.0)
Brain Based Development Disabilities	15 (7.5)
Medical Interventions	13 (6.5)
Cancer	11 (5.5)
Skin Issues	6 (3.0)
Nutritional/Metabolic Issues	5 (2.5)
Kidney Issues	3 (1.5)
Inflammatory Disease	2 (1.0)
Traumatic Injuries	2 (1.0)
Fever	1 (0.5)
Mental Health Issues	1 (0.5)
Oral Health Related Conditions	1 (0.5)
Multiple^b^	24 (12.0)
**Number of Study Participants^c^**	
1–100	70 (35.0)
101–200	37 (18.5)
201–300	27 (13.5)
301–400	10 (5.0)
401–500	2 (1.0)
>500	53 (26.5)
Median (Range)	172 (11–79,109)

**Notes**: ^a^Participants were recruited from countries spanning at least two continents. ^b^Studies included caregivers of patients that could suffer from various conditions. ^c^One study did not identify the number of participants included in their analysis.

Children and adolescents supported by caregivers suffered from a wide spectrum of conditions or diseases. The top five most prevalent conditions were infections/parasitic diseases (n = 31, 15.5%), congenital diseases (n = 23, 11.5%), gastrointestinal issues (n = 21, 10.5%), respiratory issues (n = 21, 10.5%), and diseases of the nervous system (n = 20, 10.0%). In addition, 24 included studies (12.0%) examined caregivers of children and adolescents who were not restricted to a single condition or disease.

Sample sizes of participants analyzed within the included studies varied greatly, ranging from 11 families to 79,109 caregivers. However, exact numbers of caregivers invited within the included studies is largely unknown as only 66 studies (33.0%) reported on the number of invited participants. Of note, reported response rates ranged from 16% to 100% with a median response rate of 65%.

### Economic Burden of Caregivers: Approaches and Measures

Studies used various approaches and measures to quantify and evaluate the economic burden of caregivers (Table 2). Several studies used only one approach to measure the economic burden of caregivers (n = 113, 56.5%), others used a combination of two approaches (n = 75, 37.5%), and a few used three approaches or more (n = 12, 6.0%). The three most common approaches were questionnaires/surveys (n = 182, 91.0%), interviews (n = 57, 28.5%), and medical/billing/insurance records or databases (n = 52, 26.0%). In most studies, authors developed their own questionnaires (n = 139, 69.5%), used validated scales (n = 46, 23.0%), and/or used survey data from elsewhere (n = 34, 17.0%). Another less commonly used approach was that of a cost diary (n = 8, 4.0%). Some commonly used survey data included the National Survey of Children with Special Health Care Needs (n = 13, 6.5%) and the Medical Expenditure Panel Survey (n = 7, 3.5%). Various validated scales were cited by the studies. The most used validated scales included the Work Productivity and Activity Impairment scale (n = 10, 21.7%),^252^ the Client Service Receipt Inventory (n = 3, 6.5%),^253^ and the Caregiver Impact Questionnaire (n = 3, 6.5%).^254^

**Table 2:** Approaches and Measures Used to Assess Caregivers’ Economic Burden

Study Characteristics	N (%) Total=200
**Number of different approaches used**	
1	113 (56.5)
2	75 (37.5)
3	11 (5.5)
>3	1 (0.5)
**Approaches used to measure the economic burden^a^**	
Questionnaires and Surveys	182 (91.0)
Interviews	57 (28.5)
Medical / Billing / Insurance Records or Databases	52 (26.0)
Focus Groups	3 (1.5)
Others	5 (2.5)
**Measures or scales used to assess the economic burden among those that used questionnaires or surveys^b^**	
Developed by the Investigators Conducting the Study	139 (76.4)
Previously Validated Scales	46 (25.3)
Survey Data Provided from an External Source	34 (18.7)
Cost Diary	8 (4.4)

**Notes**: ^a^Sum does not add to 200 as 87 studies used more than one approach to assess caregivers’ economic burden. ^b^Sum exceeds 100% as groups are not mutually exclusive.

### Direct Medical, Direct Non-Medical and Indirect Costs

The included studies measured various types of costs in a non-mutually exclusive manner (Table 3). More specifically, 153 studies (76.5%) measured at least some direct medical cost, 148 studies (74.0%) measured at least some direct non-medical cost, and 179 studies (89.5%) measured some indirect cost. Most studies (n = 159, 79.5%) measured cost items from more than one category and 121 studies (60.5%) measured all three types of costs. Alternatively, 10 studies (5.0%) only measured direct medical costs, three studies (1.5%) only measured direct non-medical costs and 28 studies (14.0%) only measured indirect costs. The most frequently examined cost items were those related to transportation (direct non-medical costs; n = 123, 61.5%), medication (direct medical costs; n = 117, 58.5%), and absenteeism (indirect costs; n = 115, 57.5%).

**Table 3:** List of Cost Items Considered Within the Included Studies^a^

Items	N (%) Total=200
**Direct Medical Costs**	**153 (76.5)**
Medication	117 (58.5)
Hospital Care / Services	114 (57.0)
Outpatient Care / Services	97 (48.5)
Diagnostics / Investigations / Lab Tests	64 (32.0)
Medical Devices / Materials	57 (28.5)
Out-of-Pocket Expenses^b^	56 (28.0)
Medical Transport	23 (11.5)
Other	3 (1.5)
**Direct Non-Medical Costs**	**148 (74.0)**
Transportation	123 (61.5)
Accommodations	74 (37.0)
Out-of-Pocket Expenses, unrestricted items^b^	50 (25.0)
Paid Domestic Helper	46 (23.0)
Food / Specific Diet	44 (22.0)
Informal Care	22 (11.0)
Social Services	19 (9.5)
Over-The-Counter Medication	5 (2.5)
Legal Expenses	4 (2.0)
Other	3 (1.5)
**Indirect Costs**	**179 (89.5)**
Absenteeism	115 (57.5)
Loss of Income	90 (45.0)
Changes in Work Schedule	50 (25.0)
Productivity	63 (31.5)
Loss of Leisure Time	24 (12.0)
Unspecified Time Loss	24 (12.0)
Lost Opportunities	14 (7.0)
General Financial Difficulties	11 (5.5)
Other	4 (2.0)

**Notes**: ^a^Sums can exceed 100% as studies could examine more than one cost category. ^b^Out-of-pocket expenses could be directly related to or not directly related to the child’s or adolescent’s medical needs.

### Sex and Gender Variables

Of the 200 included studies, less than half (n = 93, 46.5%) reported on the sex of the caregivers and none reported on their gender (Table 4). Twenty-seven (13.5%) of the included studies reported having explored how respondents’ sex impacted the results of their studies. However, confounding adjustment or stratification on this characteristic was only reported in 13 of these.

**Table 4:** Reporting of Caregivers’ Sex and Gender and Their Use Within Included Studies

Sex and Gender Considerations	N (%)
**Sex considerations**	
Reported on the respondents’ sex	93 (46.5)
Statistical adjustment based on respondents’ sex	24 (12.0)
Adjusted for the respondents’ sex as a confounder	5 (2.5%)^a^
Stratified on the respondents’ sex	19 (9.5)^a^
Examined how respondents’ sex impacted on the results of the study	27 (13.5)^b^
**Gender considerations**	
Reported on the respondents’ gender	0 (0.0)

**Notes**: Although certain articles reported a gender variable, we considered these as a sex variables since all of them dichotomized entries into male/female. ^a^None of the studies that accounted for respondents’ sex within their analysis used both methods. ^b^Fourteen out of the 27 studies that examined how respondents’ sex impacted their results did not account for this variable through the use of confounding adjustment or stratification.

Alternatively, of those that reported on the respondents’ sex, only 24 (12.0%) adjusted for this characteristic within their analyses; the majority (n = 19, 9.5%) of which did so by stratifying their results in function of respondents’ sex. Although sex-stratified analyses were not the focus of any of these studies, two main trends were observed. First, sex-stratified analyses were used to report how total costs differed (or did not) in function of respondents’ sex; differences or lack of were not generally discussed within the studies that examined these relations.^104^,^109^,^111^,^115^,^139^,^204^,^207^,^210^ Second, stratification on the respondents’ sex was used to account for and/or illustrate sex-based differences in indirect costs incurred by caregivers.^139^,^204^,^207^,^220^,^221^

## Discussion

Economic burden of caregivers is of particular interest for many actors, but questions remain regarding the best approaches to assess this burden. As such, we conducted a scoping review of the recent literature to examine how this burden was assessed within different groups of caregivers of child and adolescent patients. We selected 200 studies of various designs and conducted within a wide spectrum of diseases and geographical settings.^33^,^54–251^,^255^

Overall, this review highlighted that cost items examined, and approaches used to assess caregivers’ economic burden related to these items varied substantially, as some were more inclusive concerning gendered expenses. This finding was expected as it reflects the fact that the caregivers’ economic burden and the items that cause this burden will vary in function of the nature and severity of the disease or condition the patient suffers from. Nonetheless, our review still identified several interesting trends which warrant further discussion.

Researchers tend to favor combining multiple approaches and tools when assessing caregivers’ economic burden (Table 2). For example, researchers favor the use of questionnaires and surveys (n = 182, 91.0%), yet almost half of these (n = 78, 39.0%) used at least a second approach within their same study. Likewise, even if researchers clearly favored developing their own questionnaires (n = 139, 76.4%), one in four (n = 38, 19.0%) still combined these with other tools. Unfortunately, it is unclear if combining approaches and/or tools within a single study resulted in more precise or complete responses regarding this burden than selecting a single approach and/or tool.

Selected studies differed substantially in terms of cost items assessed regarding caregivers’ economic burden (Table 3). Despite these differences, our results clearly identify that researchers conducting such studies do so to assess, albeit not exclusively, respondents’ indirect costs related to caring for a child or an adolescent. We believe that this result reflects the fact that children and adolescents suffering from various diseases and conditions are likely to need external help from caregivers, therefore requiring them to dedicate paid or unpaid time to the patient. Adequately quantifying the value of the time dedicated to patients will likely require interacting with caregivers. This finding may be due to the fact that many of the studies selected focused on clinical settings where the respondent was caring for a child or an adolescent suffering from a chronic and potentially severe condition (Table 1). As previously observed by others,^24^ caregivers of such patients are not only likely to have trouble maintaining fulltime employment, the amount of time required caring for these patients will also likely be greater.

Lastly, although informal caregiving has been recognized as a sex and gender-related concept,^24^,^35^,^41–43^ most studies did not report on these considerations and those that did so tended to ignore these within their analyses and results (Table 4).

Faced with these different trends, we have identified a number of observations that may help researchers in the planning of such studies in the future. First, as previously noted, time dedicated to caring for children and adolescents suffering from various conditions and illness are frequently reported by caregivers to be related to their economic burden. Although the amount of time required to care for patients, the type of time (eg, paid work time, unpaid time) and their related cost are expected to vary in function of multiple factors, researchers should at least consider examining these within their own work. Those that did not report these cost items should explain why these were not required or examined.

Second, researchers should describe both respondents in their studies and those who were invited to participate but refused. Indeed, although the median response rate within selected study was 65%, only 66 studies (33.0%) reported this measure. Such a result raises a question regarding the validity of studies that fail to report this measure. Furthermore, seeing as included studies generally aimed to assess the monetary value of caregivers’ financial burden, this issue raises the risk that estimates provided within these studies could be biased.

Third, although not exclusively, most studies included in our review used questionnaires and surveys to assess respondents’ economic burden and investigators did so by designing their own cost questionnaires. This result likely reflects the fact that the financial impact imposed on caregivers and which cost item will be impacted could vary significantly in relation to clinical manifestation related to the patients’ condition or illness, cultural consideration and geographical settings. Nonetheless, researchers planning to assess this burden should examine the option to use previously validated scales and questionnaires (eg,^252–254^,^256^); either exclusively or to supplement them with additional targeted questions of importance to the clinical team.

Finally, sex- and gender-related characteristics have been widely omitted and/or not considered within selected studies, even though they have been shown to be related to the caregivers’ burden.^20^,^35^,^41^,^42^ Considering sex and gender with appropriate tools, following the Sex And Gender Equity in Research (SAGER) guidelines,^257^ is highly recommended. Also, as income inequity remains between females and males, it is important to account for this issue when estimated costs related to caregivers’ productivity losses. Future work could account for this issue by applying a single unit cost (eg, mean hourly wage within the sample) to the time spent providing informal caregiving.

Beyond these observations, we must recognize that our review focused solely on the financial impacts of caregiving which are but a fraction of economic considerations that could be considered within economic evaluations. Some groups have argued that broader economic aspect related to caregiver burden, such as caregivers’ spillover effects (eg, the amount of quality-adjusted life-years they have lost or gained due to caregiving), could be examined within such studies for further inclusion within subsequent health economic evaluations. Although economic evaluations combining both patient-incurred benefits (or decrements) and caregiver-incurred benefits (or decrements) can be found within the scientific literature (eg,^15^,^17^), these remain rare. While we believe that there is merit to consider caregiver spillover effects within future economic evaluation, doing so will require substantial research to adequately define how to do so. Additional research focussing on this topic is needed.

## Limitations

Our study has limitations that we must recognize. Firstly, we designed a de novo search strategy (Appendix 1) to examine caregiver economic burden within multiple literature databases. Unfortunately, caregivers and their burden remain ill-defined concepts within the scientific literature. Though we recognize that the search strategy that we proposed may be imperfect, it was designed in collaboration with a research librarian who coauthors this manuscript. Furthermore, the search strategy was independently reviewed by a second research librarian. Secondly, we categorized cost items in terms of “direct medical costs”, “direct non-medical costs” and “indirect costs”. While some authors have argued against using this categorization,^258^ we still grouped identified cost items within these three categories as they remain widely used within the scientific literature. Thirdly, some of the selected studies differentiated their respondents as “male” or “female” (eg, 19 [9.5%] studies presented sex stratified results). As sex and gender tend to be intertwined, sex is frequently considered a proxy of gender. However, gender identity goes beyond such a binary definition, as it is a spectrum.^257^ Also, gender is multifaceted. It comprises a person’s gender identity, as well as gender roles (eg, responsibility for child care), gender relationships (eg, how individuals act or are treated based on their gender), and “institutionalized gender” (eg, the way power and resources are distributed based on gender and can be reflected by education level, personal income).^259^ Therefore, sex dichotomization only partly captures gender. We encourage researchers to measure both sex and gender. We also strongly encourage researchers against using the terms sex and gender interchangeably, as this common mistake can generate confusion and even lead to incorrect interpretations and recommendations.^44^,^259^ Lastly, unlike others,^18^ we did not quantify this burden within our review and instead focused on the methods used by others to quantify this burden. Nonetheless and in addition to our observations, we provide readers with our raw structured datasets (refer to Supplementary Tables 1–4 in Appendix 3) to aid them in identifying past studies in a wide range of clinical areas and geographical locations as well as to help plan future studies aimed at assessing this burden within caregivers of child and adolescent patients.

## Conclusion

Our review identified 200 studies examining the financial burden of familial caregivers of children and adolescents. Our results highlight the wide spectrum of methods and approaches used to assess this burden but that many fail to clearly provide key data needed to evaluate the methodological quality of their work (eg, study response rates). Similarly, our results also highlighted that sex and gender-based considerations are often omitted from such studies even though caregiving is strongly related to gendered roles. Such omissions can raise concerns regarding the internal and external validity of study results. Researchers conducting such work in the future must better acknowledge methodological components of their work, including sex and gender-based considerations, to strengthen their results.
